# Early urate-lowering therapy and cardiometabolic risk in adults without documented major cardiometabolic disease: a real-world cohort study

**DOI:** 10.3389/fendo.2026.1934688

**Published:** 2026-08-31

**Authors:** An-Ping Huo, Shiow-Ing Wang, Pui-Ying Leong, James Cheng-Chung Wei, Tsai-Ching Hsu

**Affiliations:** 1 Institute of Medicine, Chung Shan Medical University, Taichung, Taiwan; 2 School of Medicine, Chung Shan Medical University, Taichung, Taiwan; 3 Division of Allergy, Immunology and Rheumatology, Department of Internal Medicine, Chung Shan Medical University Hospital, Taichung, Taiwan; 4 Center for Health Data Science, Department of Medical Research, Chung Shan Medical University Hospital, Taichung, Taiwan; 5 Department of Nursing, Chung Shan Medical University, Taichung, Taiwan; 6 Graduate Institute of Integrated Medicine, China Medical University, Taichung, Taiwan; 7 Office of Research and Development, Asia University, Taichung, Taiwan

**Keywords:** cardiometabolic risk, hyperuricemia, obesity, ROS, TriNetX, urate-lowering therapy

## Abstract

**Background:**

Whether urate-lowering therapy (ULT) affects cardiometabolic outcomes in individuals without established metabolic disease remains uncertain.

**Materials and methods:**

This retrospective cohort study used TriNetX data (2015–2023) to include adults with newly diagnosed hyperuricemia (UA >7 mg/dL) and no baseline diabetes, cardiovascular disease, or metabolic disorders. Patients initiating ULT were propensity score-matched (1:1) to untreated controls (2,906 pairs). The primary outcome was a composite cardiometabolic endpoint.

**Results:**

Over a median follow-up of 3.03 years, ULT initiation was associated with increased risk of the composite cardiometabolic outcome (HR 1.18, 95% CI 1.07–1.30) and of all-cause mortality (HR 1.52, 95% CI 1.27–1.82). The increased risk was evident among men, individuals without prior gout, users of xanthine oxidase inhibitors, and those who achieved target serum urate (<5 mg/dL) within 6 months.

**Conclusions:**

In individuals without major cardiometabolic disease, initiating ULT was associated with increased cardiometabolic risk. Serum urate may have context-dependent physiological roles in early metabolic states, warranting further research.

## Introduction

Hyperuricemia, defined as elevated serum uric acid (UA) levels, is a common metabolic abnormality and has been consistently associated with hypertension, chronic kidney disease, cardiovascular disease, insulin resistance, and metabolic syndrome (1–3). Although these epidemiological associations are well established, whether serum UA acts primarily as a causal mediator of cardiometabolic disease or reflects a physiological response to underlying metabolic stress remains uncertain.

Urate-lowering therapy (ULT) is the cornerstone of gout management; however, randomized clinical trials have not consistently demonstrated cardiovascular or metabolic benefit from pharmacological urate reduction, creating a persistent discrepancy between observational associations and interventional outcomes (4). This inconsistency suggests that the biological consequences of urate lowering may depend on disease stage, metabolic context, or the physiological role of urate itself. However, the relationship between urate and cardiometabolic health is increasingly recognized as complex and non-linear. Recent evidence highlights that not only hyperuricemia, but also extreme hypouricemia, is associated with adverse clinical presentations, suggesting that physiological urate levels must be carefully balanced (5).

Current clinical guidelines recommend ULT for patients with recurrent gout or advanced tophaceous disease but do not advocate routine treatment of asymptomatic hyperuricemia because of insufficient evidence regarding clinical benefit (6, 7). Previous studies evaluating the cardiorenal effects of ULT have largely focused on patients with established chronic kidney disease, diabetes mellitus, or overt cardiovascular disease (8, 9). Consequently, the cardiometabolic consequences of initiating ULT before the development of overt metabolic disease remain poorly understood.

To address this knowledge gap, we conducted a large real-world retrospective cohort study using a propensity score-matched electronic health record database to evaluate cardiometabolic outcomes following ULT initiation in adults with hyperuricemia but without preexisting cardiometabolic disease. By focusing on a without documented major cardiometabolic disease population, we sought to determine whether early pharmacological urate lowering is associated with subsequent cardiometabolic outcomes and to provide clinical evidence that may help elucidate the context-dependent role of urate in metabolic homeostasis.

## Research design and methods

### Study design and data source

This retrospective cohort study used data from TriNetX, a global federated research network that aggregates de-identified electronic health records from more than 250 million individuals across over 120 healthcare organizations (HCOs). TriNetX employs standardized data quality assessments for conformance, completeness, and plausibility and has been widely used in cardiovascular outcomes research. Data were extracted from the US Collaborative Network (63 HCOs) in May 2024. The study period spanned January 1, 2015, through December 31, 2023.

### Study subjects

Eligible participants were adults with newly diagnosed hyperuricemia, defined as a serum UA > 7 mg/dL documented on at least two occasions. To capture incident cases, individuals with a prior diagnosis of hyperuricemia (ICD-10-CM E79.0) or UA > 7 mg/dL before December 31, 2014, were excluded.

Patients were categorized into a ULT cohort and a control cohort. The ULT cohort included patients prescribed ULT (allopurinol, febuxostat, oxypurinol, sulfinpyrazone, lesinurad, probenecid, or benzbromarone) after the first documentation of hyperuricemia. with at least two UA measurements recorded. The index date was defined as the first ULT prescription. The control cohort comprised patients with hyperuricemia who had never received ULT, with the index date defined as the first documented hyperuricemia.

To minimize baseline confounding, patients with preexisting major cardiometabolic diseases were excluded. Specifically, we applied a 1-year look-back period prior to the index date to exclude patients with any documented history of circulatory system diseases (ICD-10-CM: I00-I99), diabetes mellitus (E08-E13), abnormal glucose or hyperglycemia (R73.0, R73.9, R81, R82.4), and disorders of lipoprotein metabolism (E78), as well as those prescribed cardiac therapy or anti-hypertensive/lipid-lowering medications (ATC codes C01, C02, C07, C03, C08, C09, C10A, A10). We intentionally retained obesity (ICD-10-CM: E66) and chronic kidney disease (ICD-10-CM: N18) as baseline covariates rather than exclusion criteria. This approach was chosen because these conditions are highly prevalent in patients with hyperuricemia and reflect the real-world baseline risk profile of this population, while still allowing us to investigate the incidence of new, overt cardiometabolic events. (Detailed code lists are provided in **Supplementary Table 1**). Following an ‘as-started’ (intention-to-treat analog) design, patients were retained in their respective baseline cohorts and followed continuously until an outcome event, death, or study end, regardless of subsequent medication adherence, discontinuation, or crossover (e.g., controls who later received ULT).

### Outcomes

The primary outcome was a composite cardiometabolic endpoint encompassing including essential hypertension (ICD-10: I10), ischemic heart disease (I20-I25), cerebral infarction (I63), and dyslipidemia (E78). This composite endpoint was chosen because these conditions collectively reflect the interconnected continuum of early metabolic deterioration and atherosclerotic cardiovascular disease (ASCVD). In a population without prior cardiometabolic disease, a composite measure provides a comprehensive assessment of overall metabolic risk. The specific ICD-10 codes for all outcomes are detailed in **Supplementary Table 1**. The secondary outcome was all-cause mortality. To minimize protopathic bias, the follow-up for outcome assessment strictly started 3 months after the index date and extended up to a maximum of 7 years.

### Covariates

Baseline covariates were assessed within one year before the index date and included demographics, socioeconomic status, tobacco and alcohol use disorders, healthcare utilization metrics, comorbidities (obesity, chronic kidney disease, gout), and laboratory measures (serum UA, BMI, estimated glomerular filtration rate).

### Statistical analyses

Propensity score matching (1:1 nearest neighbor, caliper 0.1 SD) was performed using predefined covariates. Covariate balance was assessed using standardized mean differences (<0.1 indicating adequate balance). Time-to-event analyses were conducted using Kaplan–Meier methods and Cox proportional hazards models, with proportional hazards assumptions tested. Prespecified subgroup and sensitivity analyses are detailed in the Supplement. The primary and secondary outcomes were evaluated from the end of the 3-month landmark period until the occurrence of the event. Patients who did not experience the outcome were right-censored at the time of death, the maximum 7-year follow-up limit, or the end of the database extraction period (December 31, 2023), whichever occurred first. This yielded a median follow-up of 3.03 years (mean: 3.86 years; maximum: 7.00 years) for the matched cohort.

Because patients in the ULT cohort were categorized based on their initial prescription and followed continuously regardless of subsequent medication adherence, discontinuation, or cross-over, our analytical framework inherently adopted an ‘as-started’ approach, which serves as the observational analog to an intention-to-treat (ITT) analysis.

To quantify the potential impact of unmeasured confounding on our primary findings, we calculated the E-value for the primary composite outcome. The E-value estimates the minimum strength of association that an unmeasured confounder would need to have with both the treatment and the outcome to fully explain away the observed association.

Furthermore, while we attempted to adjust for baseline differences, alternative explanations for the observed associations must be considered. These include reverse causation (where underlying, undiagnosed cardiometabolic deterioration prompts clinical actions that lead to ULT initiation), diagnostic surveillance bias (although baseline healthcare utilization was adjusted for), and incomplete capture of mortality events that occurred outside the participating healthcare organizations.

## Results

**Figure 1:**
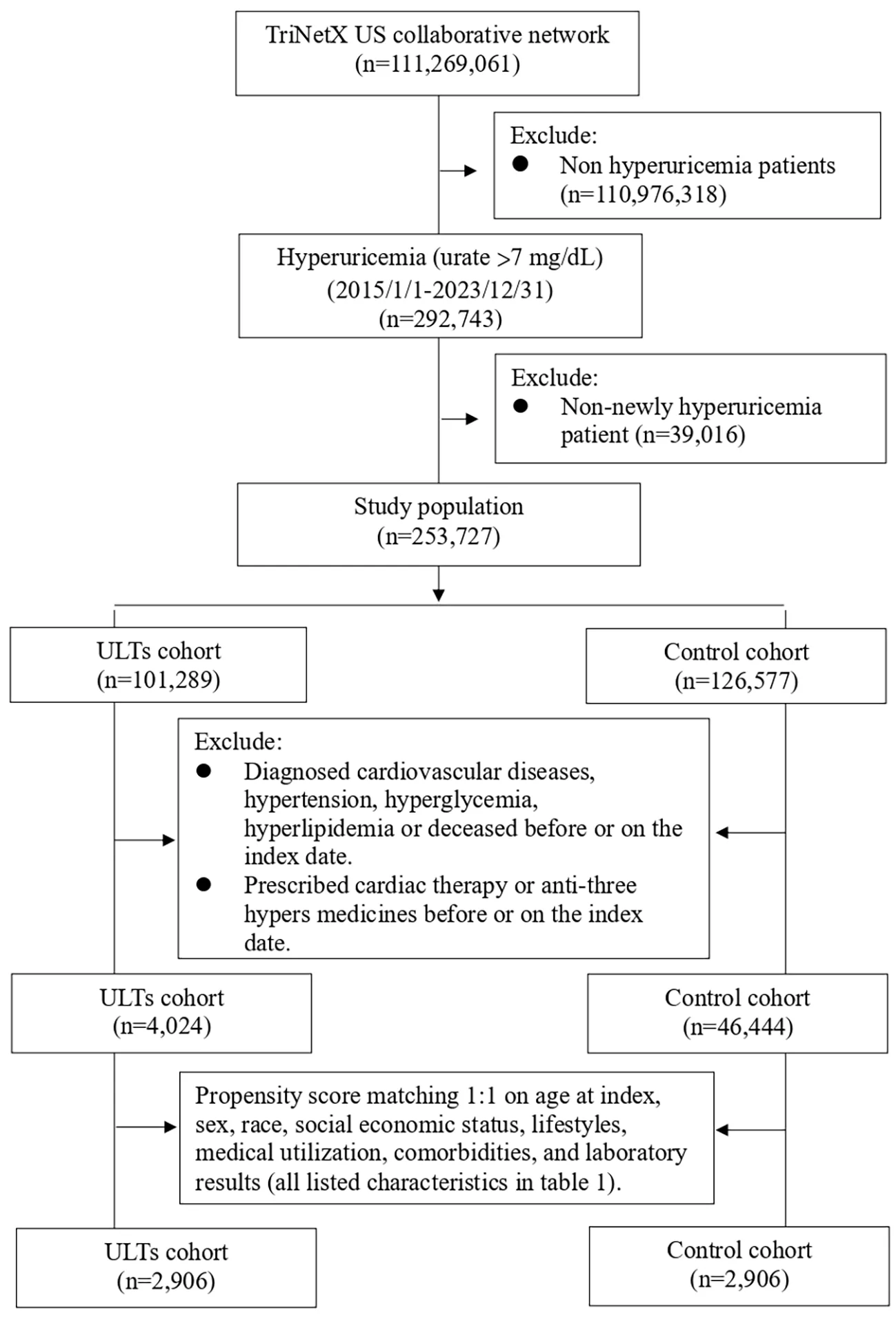
Selection Process.

After propensity score matching, 2,906 patients were included in each cohort (**Figure 1**). Baseline characteristics were well balanced after matching, with standardized mean differences(SMDs) <0.1 for all variables except chronic kidney disease (**Supplementary Table 2**). Notably, the residual imbalance in chronic kidney disease (SMD = 0.121) was driven by a slightly higher prevalence in the untreated control cohort (6.9%) compared to the ULT cohort (4.1%).”

**Figure 2:**
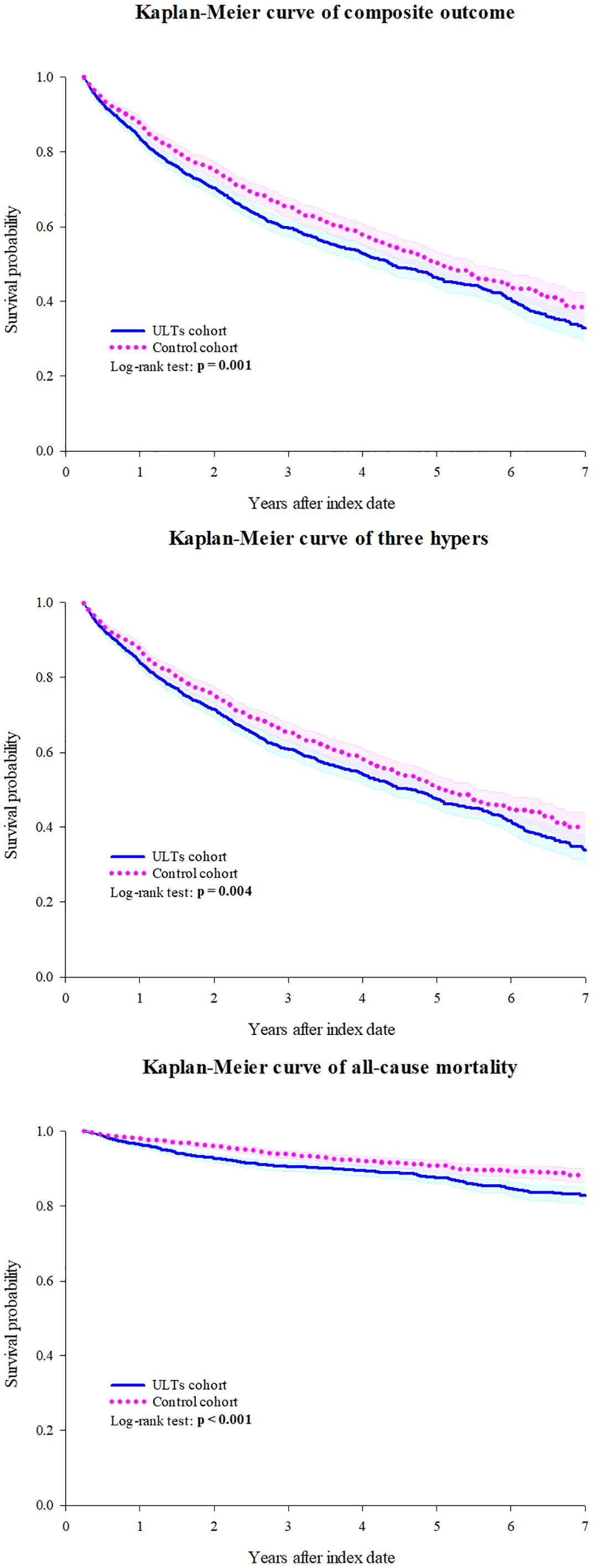
**(a)** Kaplan-Meier curve of composite outcome between the urate-lowering therapy group and the control group. **(b)** Kaplan-Meier curve of dyslipidemia-related conditions between the urate-lowering therapy group and the control group. **(c)** Kaplan-Meier curve of all-cause mortality between the urate-lowering therapy group and the control group.

During follow-up, initiation of ULT was associated with a higher incidence of the composite cardiometabolic outcome (HR 1.18; 95% CI, 1.07–1.30), dyslipidemia-related conditions (HR 1.15; 95% CI, 1.05–1.27), and all-cause mortality (HR 1.52; 95% CI, 1.27–1.82) (**Table 1**). Kaplan–Meier curves demonstrated higher cumulative incidence of these outcomes in the ULT cohort (**Figures 2a–c**).

**Table 1:** Risk of outcomes (3 months to 7 years).

Outcomes	Patients with outcome		Hazard ratio (PSM-cohort) (95% CI) ^a^
	ULTs cohort (n=2906)	Control cohort (n=2906)	
**Composite outcome**	1016	644	**1.179 (1.068-1.301)**
Essential Hypertension	599	437	1.090 (0.963-1.233)
Cerebral infarction, unspecific	34	41	0.842 (0.535-1.327)
Ischemic heart disease	173	185	0.893 (0.726-1.099)
Dyslipidemia-related conditions	992	641	**1.154 (1.045-1.274)**
**All-cause mortality**	286	193	**1.519 (1.266-1.824)**

ULTs, Urate-lowering therapeutics; CI, Confidence interval; NA, Not available.

If the patient is less or equal to 10, results show the count as 10.

a. Propensity score matching was performed on all listed characteristics.

b. Incidence rates and person-years are not explicitly exported by the TriNetX platform’s survival module. The Hazard Ratios were calculated internally by the system’s Cox proportional hazards model, which intrinsically accounts for differential follow-up times, person-years at risk, and censoring events between the matched cohorts. Bold value mead increased Hazard Ratio.

Subgroup and sensitivity analyses demonstrated heterogeneity by sex, gout history, ULT mechanism, degree of urate reduction, and timing of therapy initiation (**Supplementary Tables S3**–**S11**).

## Discussion

In this large real-world cohort of adults with hyperuricemia but no prior cardiometabolic disease, initiation of ULT was associated with an increased risk of subsequent cardiometabolic outcomes and all-cause mortality. These associations were more pronounced among men, individuals without prior gout, and those initiating treatment early or achieving rapid urate reduction. By focusing on a without documented major cardiometabolic disease population, our findings address an important evidence gap regarding the cardiometabolic consequences of early urate-lowering strategies.

Several observations support the robustness of these findings. First, healthcare utilization was not higher in the treated cohort, making surveillance bias unlikely. Second, the observed associations were consistent across age groups and baseline serum urate levels. Third, the increased risk was confined to individuals without a history of gout and was absent among those with established gouty arthritis, suggesting that the clinical context of urate lowering may be important. Furthermore, subgroup analyses showed differential associations by ULT class: xanthine oxidase inhibitors were associated with increased risk, whereas uricosuric agents were not. Sensitivity analyses further demonstrated that early treatment initiation and target urate achievement were associated with higher cardiometabolic risk.

The biological explanation for these observations remains uncertain. Although hyperuricemia has traditionally been viewed as a pathogenic contributor to cardiometabolic disease, accumulating evidence suggests that serum urate may have context-dependent physiological functions. Humans lost functional uricase during evolution, resulting in substantially higher circulating urate concentrations than in most mammals (10). In extracellular environments, uric acid functions as an important endogenous antioxidant, whereas excessive intracellular urate accumulation may promote oxidative stress and inflammation (11). These dual biological properties raise the possibility that the metabolic consequences of pharmacologic urate reduction may vary by disease stage and the underlying oxidative milieu. Indeed, the clinical implications of serum urate are highly context-dependent rather than uniformly linear. Clinical studies evaluating emergency department populations have demonstrated the distinct clinical significance of hypouricemia (5), while other studies have highlighted the complex, independent associations between serum uric acid levels and acute cerebrovascular events such as stroke (12). These observations strongly support the premise that blindly aggressively lowering urate without considering the patient’s baseline metabolic and redox context may yield unintended cardiometabolic consequences.

Visceral adiposity and oxidative stress are increasingly recognized as central drivers of insulin resistance and cardiometabolic dysfunction (13, 14). Serum urate has been shown to correlate with visceral adiposity and markers of systemic oxidative stress, suggesting that elevated urate in early metabolic states may reflect a compensatory response rather than a purely pathogenic process (15–22). However, whether early pharmacologic urate reduction alters this balance remains speculative and requires further mechanistic validation.

**Figure 3:**
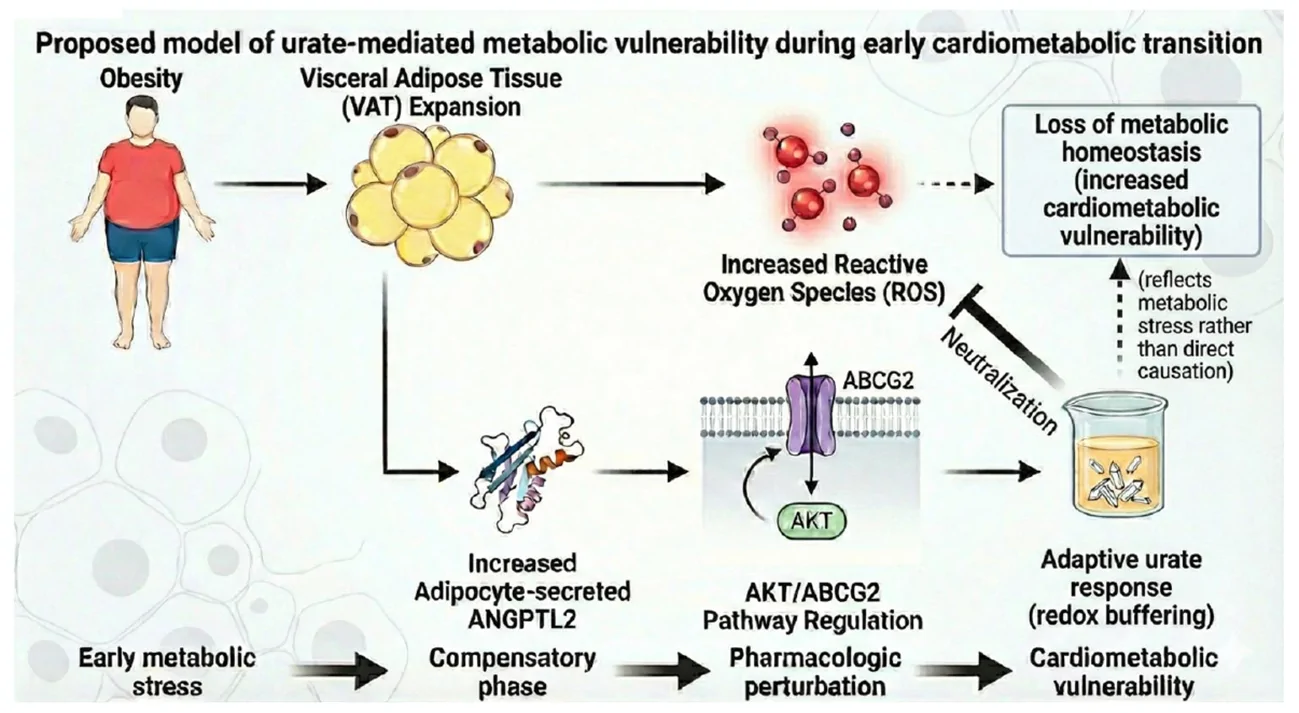
Proposed model of urate-mediated metabolic vulnerability during early cardiometabolic transition. The mechanisms proposed in this image are speculative and purely hypothesis-generating. In without documented major cardiometabolic disease individuals, early metabolic stress—particularly linked to visceral adiposity—increases oxidative burden and triggers an adaptive rise in serum urate, which may serve as a redox-buffering response rather than a direct pathogenic mediator. Pharmacologic urate-lowering therapy, especially when it abruptly suppresses endogenous urate production via xanthine oxidase inhibition, may perturb this adaptive equilibrium. Disruption of urate-mediated redox buffering in early disease stages may reduce metabolic resilience and increase vulnerability to subsequent cardiometabolic dysfunction. This model is hypothesis-generating and intended to illustrate a testable disease trajectory rather than establish causality.

From a pathophysiological perspective, serum urate is more strongly associated with visceral adipose tissue (VAT) than with subcutaneous adipose tissue and independently correlates with impaired glucose metabolism (23–25). As VAT expands, systemic oxidative stress rises, triggering compensatory antioxidant responses, including increased plasma ferric-reducing ability and catalase activity, both of which are positively associated with circulating urate levels (26). Moreover, visceral obesity–related adipokines such as ANGPTL2 may further promote hyperuricemia via renal tubular AKT/ABCG2 signaling (27).Taken together, these observations provide a biologically plausible framework in which elevated serum urate during early metabolic dysfunction may represent a compensatory response to increased oxidative stress. Whether pharmacologic urate lowering modifies this adaptive response requires further mechanistic investigation (**Figure 3**).

Although ULT remains the standard treatment for gout, its role in asymptomatic hyperuricemia remains controversial. Our findings suggest that the metabolic consequences of urate lowering may depend on disease stage and patient characteristics. These observations underscore the need to consider the timing and clinical context of ULT initiation when evaluating individuals without established cardiometabolic disease.

This study has several strengths, including a large real-world cohort, propensity score matching, and multiple sensitivity analyses to minimize confounding and protopathic bias. However, several limitations should be acknowledged. Residual confounding and confounding by indication cannot be fully ruled out despite extensive propensity score matching and sensitivity analyses. Additionally, exposure in this study was defined by electronic prescription records. Because the TriNetX platform does not capture pharmacy dispensation data or exact adherence metrics, we could not confirm whether medications were actually consumed, nor could we reliably perform ‘as-treated’ or time-varying exposure analyses. However, our ‘as-started’ design provides a conservative estimate of real-world clinical effectiveness. Additionally, mechanistic pathways linking urate reduction to cardiometabolic outcomes were not directly assessed. Prospective studies and mechanistic investigations are needed to clarify whether the observed associations reflect causal biological effects or unmeasured confounding.

Finally, several critical methodological limitations inherent to the federated nature of the TriNetX platform must be acknowledged. First, the platform’s strict privacy protocols prevent the extraction of patient-level raw data, which precludes the use of advanced epidemiological designs—such as emulating a common time zero or implementing time-varying covariates—to fully eliminate immortal-time bias. Second, the built-in statistical modules do not support competing risk models, such as Fine-Gray or cause-specific Cox regressions, which are ideally suited for populations with high baseline mortality. Furthermore, the system lacks the capability to perform time-dependent exposure models to evaluate post-baseline urate changes without immortal-time bias, nor can it calculate formal interaction P-values for subgroup comparisons, adjust for multiple testing, perform strict intention-to-treat analyses, or account for clustering effects across different healthcare organizations. Consequently, these findings should be considered hypothesis-generating and warrant further exploration and confirmation through prospective studies or databases that permit robust, patient-level raw data analysis.

Another important limitation is the potential for residual confounding by indication, a common challenge in observational pharmacoepidemiology. For instance, a slight imbalance in chronic kidney disease remained after propensity score matching. However, because this condition was actually more prevalent in the untreated control cohort, this baseline disadvantage would theoretically bias the results toward the null, thereby reinforcing the robustness of our finding that ULT is associated with higher risk. Additionally, our E-value analysis quantitatively demonstrated that only a relatively strong unmeasured confounder (risk ratio ≥ 1.63) could fully explain away the observed primary association.

## Conclusion

In without documented major cardiometabolic disease individuals with hyperuricemia, initiating ULT was associated with increased cardiometabolic risk and all-cause mortality, particularly among those without prior gout and among those receiving early treatment or experiencing rapid urate reduction. These findings suggest that the physiological consequences of pharmacologic urate lowering may depend on metabolic context and disease stage, supporting the hypothesis that serum urate may have context-dependent roles in metabolic homeostasis. Further prospective clinical studies and mechanistic investigations are needed to determine the biological mechanisms underlying these associations.

## Data Availability

The original contributions presented in the study are included in the article/**Supplementary Material**. Further inquiries can be directed to the corresponding authors.
