# Supplementary material for: Early urate-lowering therapy and cardiometabolic risk in adults without documented major cardiometabolic disease: a real-world cohort study
Source: Front Endocrinol (Lausanne). 2026 Aug 31;17:1934688. doi: 10.3389/fendo.2026.1934688 (PMC13572265; doi:10.3389/fendo.2026.1934688)
Supplement: Supplementary file 1 [file Table1.docx]

**Supplementary materials**

**Supplementary Table S1** Codes for the present study.

| **Variables** | **Codes** |
| --- | --- |
| **Study subject** |  |
| Serum uric acid (Urate in serum or plasma) | TNX 9071 |
| Hyperuricemia | ICD-10-CM: E79.0 |
| Circulatory system disease | ICD-10-CM: I00-I99 |
| Diabetes mellitus | ICD-10-CM: E08-E13 |
| Abnormal glucose | ICD-10-CM: R73.0 |
| Unspecified hyperglycemia | ICD-10-CM: R73.9 |
| Glycosuria | ICD-10-CM: R81 |
| Acetonuria | ICD-10-CM: R82.4 |
| Disorders of lipoprotein metabolism | ICD-10-CM: E78 |
| Cardiac therapy medicines | ATC: C01 |
| Cardiometabolic medications (Anti-hypertensive, lipid-lowering, anti-diabetic) | ATC: C02, C07, C03AA, C03BA, C03CA, C03DA, C08CA, C08DA, C08DB, C09AA, C09CA, C10A, A10 |
| Allopurinol | ATC: M04AA01 |
| Febuxostat | ATC: M04AA03 |
| Sulfinpyrazone | ATC: M04AB02 |
| Lesinurad | ATC: M04AB05 |
| Probenecid | ATC: M04AB01 |
| Benzbromarone | ATC: M04AB03 |
| Oxypurinol | RXNORM: OMOP5175234 |
| **Outcomes** |  |
| Essential hypertension | ICD-10-CM: I10 |
| Cerebral infarction | ICD-10-CM: I63.9 |
| Ischemic heart disease | ICD-10-CM: I20-I25 |
| Dyslipidemia-related conditions | ICD-10-CM: I10-I1A, I67.4, E08-E13, R73.0、R73.9、R81、R82.4, E78 |
| **Covariates** |  |
| **Demographic covariates** |  |
| Social economic status (SES, problems related to housing and economic circumstances) | ICD-10-CM: Z59 |
| **Lifestyle-related variables** |  |
| Tobacco use | ICD-10-CM: Z72.0 |
| Nicotine dependence | ICD-10-CM: F17 |
| Alcohol related disorders | ICD-10-CM: F10 |
| **Medical utilization** |  |
| Office or other outpatient services | CPT: 1013626 |
| Emergency department services | CPT: 1013711 |
| Hospital inpatient services | CPT: 1013659 |
| Preventive medicine services | CPT: 1013829 |
| **Comorbidities** |  |
| Overweight and obesity | ICD-10-CM: E66 |
| Chronic kidney disease | ICD-10-CM: N18 |
| Gout | ICD-10-CM: M10 |
| **Laboratory measurements** |  |
| Body mass index | TNX curated: 9083 |
| eGFR | TNX curated: 8001 |

Note:

ICD-10-CM: International Classification of Diseases, Tenth Revision, Clinical Modification;

CPT: Current Procedural Terminology;

ATC: Anatomical Therapeutic Chemical Classification System;

TNX: TriNetX;

OMOP: Observational Medical Outcomes Partnership;

eGFR: Glomerular filtration rate/1.73 m^2^ predicted in serum, plasma by creatinine-based formula.

Table S2 Baseline characteristics of study subjects. (before and after matching)

|  | Before PSM | | | After PSM^a^ | | |
| --- | --- | --- | --- | --- | --- | --- |
| **Variables** | ULTs cohort  (n=4024) | Control cohort  (n=46444) | SMD | ULTs cohort  (n=2906) | Control cohort  (n=2906) | SMD |
| **Age at Index** |  |  |  |  |  |  |
| Mean ± SD | 48.9 ± 15.7 | 55.3 ± 19.0 | **0.368** | 49.3 ± 16.3 | 50.1 ± 18.0 | 0.045 |
| **Sex, n (%)** |  |  |  |  |  |  |
| Male | 3345 (83.1) | 26429 (56.9) | **0.597** | 2317 (79.7) | 2321 (79.9) | 0.003 |
| Female | 460 (11.4) | 17624 (37.9) | **0.646** | 425 (14.6) | 445 (15.3) | 0.019 |
| Unknown Gender | 219 (5.4) | 2391 (5.1) | 0.013 | 164 (5.6) | 140 (4.8) | 0.037 |
| **Race, n (%)** |  |  |  |  |  |  |
| White | 2602 (64.7) | 27894 (60.1) | 0.095 | 1862 (64.1) | 1906 (65.6) | 0.032 |
| Black or African American | 419 (10.4) | 6873 (14.8) | **0.132** | 319 (11.0) | 323 (11.1) | 0.004 |
| Asian | 293 (7.3) | 2159 (4.6) | **0.111** | 201 (6.9) | 194 (6.7) | 0.010 |
| Other Race | 180 (4.5) | 1643 (3.5) | 0.048 | 136 (4.7) | 138 (4.7) | 0.003 |
| Native Hawaiian or Other Pacific Islander | 35 (0.9) | 297 (0.6) | 0.027 | 18 (0.6) | 17 (0.6) | 0.004 |
| American Indian or Alaska Native | 10 (0.2) | 90 (0.2) | 0.012 | 10 (0.3) | 10 (0.3) | 0.000 |
| Unknown Race | 490 (12.2) | 7488 (16.1) | **0.113** | 366 (12.6) | 323 (11.1) | 0.046 |
| **Social economic status, n (%)** |  |  |  |  |  |  |
| Problems related to housing and economic circumstances | 13 (0.3) | 124 (0.3) | 0.010 | 13 (0.4) | 10 (0.3) | 0.016 |
| **Lifestyles, n (%)** |  |  |  |  |  |  |
| Nicotine dependence | 113 (2.8) | 2049 (4.4) | 0.086 | 92 (3.2) | 100 (3.4) | 0.015 |
| Alcohol related disorders | 49 (1.2) | 833 (1.8) | 0.047 | 41 (1.4) | 44 (1.5) | 0.009 |
| Tobacco use | 31 (0.8) | 503 (1.1) | 0.033 | 25 (0.9) | 28 (1.0) | 0.011 |
| **Medical utilization, n (%)** |  |  |  |  |  |  |
| Office or Other Outpatient Services | 1713 (42.6) | 17266 (37.2) | **0.110** | 1256 (43.2) | 1273 (43.8) | 0.012 |
| Preventive Medicine Services | 465 (11.6) | 2241 (4.8) | **0.247** | 340 (11.7) | 314 (10.8) | 0.028 |
| Hospital Inpatient and Observation Care Services | 268 (6.7) | 6729 (14.5) | **0.257** | 259 (8.9) | 272 (9.4) | 0.016 |
| Emergency Department Services | 266 (6.6) | 8130 (17.5) | **0.339** | 246 (8.5) | 297 (10.2) | 0.060 |
| **Comorbidities, n (%)** |  |  |  |  |  |  |
| Gout | 1325 (32.9) | 3143 (6.8) | **0.694** | 889 (30.6) | 885 (30.5) | 0.003 |
| Overweight and obesity | 258 (6.4) | 5473 (11.8) | **0.188** | 215 (7.4) | 212 (7.3) | 0.004 |
| Chronic kidney disease (CKD) | 136 (3.4) | 9158 (19.7) | **0.529** | 120 (4.1) | 200 (6.9) | **0.121** |
| **Laboratories, n (%)** |  |  |  |  |  |  |
| Urate in Serum or Plasma (mg/dL) |  |  |  |  |  |  |
| > 7~ < 10 | 2342 (58.2) | 42527 (91.6) | **0.833** | 2301 (79.2) | 2262 (77.8) | 0.033 |
| ≧10 | 795 (19.8) | 4021 (8.7) | **0.322** | 667 (23.0) | 651 (22.4) | 0.013 |
| eGFR (mL/min/1.73m^2^) |  |  |  |  |  |  |
| < 60 | 891 (22.1) | 23785 (51.2) | **0.633** | 843 (29.0) | 856 (29.5) | 0.010 |
| 60~ < 90 | 1577 (39.2) | 23539 (50.7) | **0.233** | 1392 (47.9) | 1398 (48.1) | 0.004 |
| ≧90 | 887 (22.0) | 14320 (30.8) | **0.200** | 808 (27.8) | 788 (27.1) | 0.015 |
| BMI (kg/m^2^) |  |  |  |  |  |  |
| ≧30 | 1076 (26.7) | 11469 (24.7) | 0.047 | 815 (28.0) | 838 (28.8) | 0.018 |

Note:

PSM: propensity score matching, ULTs: Urate-lowering therapeutics, SMD: standardized mean difference, SD: standard deviation, eGFR: Glomerular filtration rate/1.73 sq M. predicted in serum, plasma or blood by creatinine-based formula, BMI: Body mass index.

* Propensity score matching was performed on all listed characteristics.

Table S3. Risk of outcomes, adjusted for different variables. (3 months to 7 years)

|  | **Hazard ratio (95% CI)** | | | |
| --- | --- | --- | --- | --- |
| **Outcomes**  (ULTs vs. Control cohort) | **Model 1^a^** | **Model 2^b^** | **Model 3^c^** | **Model 4^d^**  **(original)** |
| **Composite outcome** | **1.455 (1.378-1.536)** | **1.323 (1.196-1.462)** | **1.225 (1.107-1.356)** | **1.179 (1.068-1.301)** |
| Essential Hypertension | 1.039 (0.970-1.113) | 1.070 (0.947-1.209) | 1.045 (0.922-1.183) | 1.090 (0.963-1.233) |
| Cerebral infarction, unspecific | **0.460 (0.342-0.618)** | 0.785 (0.505-1.221) | 0.675 (0.436-1.043) | 0.842 (0.535-1.327) |
| Ischemic heart disease | **0.631 (0.556-0.715)** | **0.811 (0.660-0.996)** | 0.883 (0.715-1.090) | 0.893 (0.726-1.099) |
| Three hypers | **1.442 (1.365-1.523)** | **1.289 (1.165-1.426)** | **1.186 (1.072-1.312)** | **1.154 (1.045-1.274)** |
| **All-cause mortality** | **0.708 (0.635-0.789)** | 1.156 (0.972-1.375) | **1.198 (1.006-1.428)** | **1.519 (1.266-1.824)** |

Note:

ULTs: Urate-lowering therapeutics, CI: Confidence interval. NA: Not available.

a. Crude, before matching.

b. Propensity score matching was performed on age at index, sex, race, and urate.

c. Propensity score matching was performed on age at index, sex, race, socioeconomic status, lifestyles, medical utilization, and urate.

d. Propensity score matching was performed on all listed characteristics

* Proportionality <0.001.

Table S4. Risk of outcomes by different follow-up durations.

|  | **Hazard ratio (95% CI)**^a^ | | | |
| --- | --- | --- | --- | --- |
| **Outcomes**  (ULTs vs. Control cohort) | **3 m to 1y** | **3 m to 3y** | **3 m to 5y** | **3 m to 7y** |
| **Composite outcome** | **1.358 (1.141-1.617)** | **1.224 (1.093-1.371)** | **1.178 (1.064-1.306)** | **1.179 (1.068-1.301)** |
| Essential Hypertension | 1.230 (0.986-1.535) | 1.084 (0.939-1.252) | 1.054 (0.927-1.198) | 1.090 (0.963-1.233) |
| Cerebral infarction, unspecific | 0.766 (0.285-2.058) | 0.665 (0.373-1.187) | 0.660 (0.395-1.104) | 0.842 (0.535-1.327) |
| Ischemic heart disease | 1.399 (0.913-2.145) | 0.928 (0.719-1.197) | 0.938 (0.750-1.173) | 0.893 (0.726-1.099) |
| Three hypers | **1.306 (1.097-1.554)** | **1.181 (1.054-1.324)** | **1.144 (1.032-1.268)** | **1.154 (1.045-1.274)** |
| **All-cause mortality** | **1.886 (1.339-2.656)** | **1.630 (1.315-2.020)** | **1.480 (1.221-1.793)** | **1.519 (1.266-1.824)** |

Note:

ULTs: Urate-lowering therapeutics, CI: Confidence interval. NA: Not available.

a. Propensity score matching was performed on all listed characteristics.

Table S5. Risk of outcomes (3 months to 7 years) stratified by urate level.

|  | **Adjusted hazard ratio (95% CI) ^a^** | | | |
| --- | --- | --- | --- | --- |
| **Outcomes**  (ULTs vs. Control cohort) | **Urate**  **(7.00~7.99 mg/dL)^b^**  (n=459 pairs) | **Urate**  **(8.00~8.99 mg/dL)^c^**  (n=309 pairs) | **Urate**  **(9.00~9.99 mg/dL)^d^**  (n=197 pairs) | **Urate**  **(≧ 10 mg/dL)^e^**  (n=288 pairs) |
| **Composite outcome** | **1.513 (1.166-1.963)** | **1.669 (1.191-2.339)** | 1.180 (0.772-1.806) | **1.709 (1.170-2.496)** |
| Essential Hypertension | 1.322 (0.950-1.839) | **1.768 (1.168-2.677)** | 0.906 (0.546-1.502) | 1.521 (0.982-2.354) |
| Cerebral infarction, unspecific | 0.933 (0.300-2.895) | 0.895 (0.125-6.382) | 0.302 (0.031-2.910) | 0.612 (0.173-2.171) |
| Ischemic heart disease | 1.516 (0.885-2.597) | 0.736 (0.421-1.286) | 1.181 (0.439-3.173) | 0.671 (0.310-1.451) |
| Three hypers | **1.479 (1.139-1.920)** | **1.604 (1.143-2.251)** | 1.172 (0.771-1.783) | **1.600 (1.102-2.325)** |
| **All-cause mortality** | **1.730 (1.050-2.851)** | 1.637 (0.842-3.184) | 1.853 (0.785-4.372) | 1.100 (0.692-1.749) |

Note:

ULTs: Urate-lowering therapeutics, CI: Confidence interval. NA: Not available.

a. Propensity score matching was performed on all listed characteristics.
b. During the study period, the subject's urate levels were recorded as between 7.00 and 7.99 mg/dL on at least two occasions, and there were no instances where urate levels reached or exceeded 8 mg/dL.
c. During the study period, the subject's urate levels were recorded as between 8.00 and 8.99 mg/dL on at least two occasions, and there were no instances where urate levels reached or exceeded 9 mg/dL.
d. During the study period, the subject's urate levels were recorded as between 9.00 and 9.99 mg/dL on at least two occasions, and there were no instances where urate levels reached or exceeded 10 mg/dL.
e. During the study period, the subject's urate levels were equal or exceeded 10 mg/dL.

Table S6. Risk of outcomes (3 months to 7 years) stratified by sex.

|  | **Adjusted hazard ratio (95% CI) ^a^** | |
| --- | --- | --- |
| **Outcomes**  **(**ULTs cohort vs. Control cohort) | **Male**  (n=2358 pairs) | **Female**  (n=374 pairs) |
| **Composite outcome** | **1.142 (1.024-1.274)** | 1.165 (0.850-1.597) |
| Essential Hypertension | 1.123 (0.976-1.291) | 0.901 (0.632-1.282) |
| Cerebral infarction, unspecific | 0.689 (0.410-1.158) | 0.478 (0.147-1.556) |
| Ischemic heart disease | 0.879 (0.691-1.117) | **0.538 (0.324-0.895)** |
| Three hypers | **1.120 (1.004-1.250)** | 1.181 (0.858-1.626) |
| **All-cause mortality** | **1.280 (1.036-1.580)** | **1.626 (1.133-2.334)** |

Note:

ULTs: Urate-lowering therapeutics, CI: Confidence interval. NA: Not available.

^a.^ Propensity score matching was performed on all listed characteristics.

Table S7. Risk of outcomes (3 months to 7 years) stratified by age at index.

|  | **Adjusted hazard ratio (95% CI) ^a^** | | |
| --- | --- | --- | --- |
| **Outcomes**  **(**ULTs cohort vs. Control cohort) | **18~39y**  (n=890 pairs) | **40~64y**  (n=2096 pairs) | **≧ 65y**  (n=2524 pairs) |
| **Composite outcome** | **1.356 (1.120-1.642)** | **1.230 (1.082-1.398)** | **1.314 (1.083-1.595)** |
| Essential Hypertension | 1.306 (0.991-1.721) | **1.177 (1.008-1.373)** | **1.310 (1.079-1.591)** |
| Cerebral infarction, unspecific | 1.003 (0.141-7.118) | 0.774 (0.424-1.413) | 0.821 (0.582-1.159) |
| Ischemic heart disease | 0.717 (0.360-1.431) | 1.090 (0.835-1.423) | **1.221 (1.017-1.466)** |
| Three hypers | **1.334 (1.102-1.614)** | **1.201 (1.056-1.365)** | **1.275 (1.050-1.550)** |
| **All-cause mortality** | **2.618 (1.446-4.739)** | **1.338 (1.037-1.725)** | **1.279 (1.088-1.504)** |

Note:

ULTs: Urate-lowering therapeutics, CI: Confidence interval. NA: Not available.

^a.^ Propensity score matching was performed on all listed characteristics.

Table S8. Risk of outcomes (3 months to 7 years) stratified by gout history.

|  | **Adjusted hazard ratio (95% CI) ^a^** | |
| --- | --- | --- |
| **Outcomes**  **(**ULTs cohort vs. Control cohort) | **With gout history^b^**  (n=726 pairs) | **Without gout history^b^**  (n=1921 pairs) |
| **Composite outcome** | 1.003 (0.856-1.176) | **1.241 (1.091-1.412)** |
| Essential Hypertension | 1.246 (0.999-1.554) | 1.085 (0.929-1.268) |
| Cerebral infarction, unspecific | 0.895 (0.223-3.583) | 1.023 (0.597-1.754) |
| Ischemic heart disease | 1.297 (0.804-2.093) | **0.720 (0.564-0.919)** |
| Three hypers | 1.003 (0.855-1.176) | **1.217 (1.069-1.387)** |
| **All-cause mortality** | 1.049 (0.499-2.206) | **1.608 (1.313-1.969)** |

Note:

ULTs: Urate-lowering therapeutics, CI: Confidence interval. NA: Not available.

a. Propensity score matching was performed on all listed characteristics.
b. Defined by ICD-10-CM code M10 occurred within 1 year on or before the index date.

Table S9. Risk of outcomes (3 months to 7 years) stratified by ULTs medication.

|  | **Adjusted hazard ratio (95% CI) ^a^** | |
| --- | --- | --- |
| **Outcomes**  **(**ULTs cohort vs. Control cohort) | **Inhibiting uric acid production^b^**  (n= 2723 pairs) | **Increasing uric acid excretion^c^**  (n= 34 pairs) |
| **Composite outcome** | **1.189 (1.072-1.320)** | 1.998 (0.766-5.207) |
| Essential Hypertension | 1.133 (0.996-1.289) | 1.815 (0.567-5.811) |
| Cerebral infarction, unspecific | 0.680 (0.440-1.049) | NA |
| Ischemic heart disease | **0.747 (0.603-0.925)** | 0.504 (0.111-2.282) |
| Three hypers | **1.157 (1.043-1.285)** | 1.998 (0.766-5.207) |
| **All-cause mortality** | **1.361 (1.129-1.641)** | 0.780 (0.107-5.706) |

Note:

ULTs: Urate-lowering therapeutics, CI: Confidence interval. NA: Not available.

a. Propensity score matching was performed on all listed characteristics.
b. Including allopurinol, febuxostat, or oxypurinol.
c. Including sulfinpyrazone, benzbromarone, lesinurad, or probenecid.

Table S10. Sensitivity analysis focus on urate level change within 6 months after the index date

|  | **Adjusted hazard ratio (95% CI) ^a^** | |
| --- | --- | --- |
| **Outcomes**  **(**ULTs cohort vs. Control cohort) | **Urate dropped ^b^**  (n=483 pairs) | **Urate not dropped ^c^**  (n=1978 pairs) |
| **Composite outcome** | **1.391 (1.061-1.823)** | 1.113 (0.991-1.251) |
| Essential Hypertension | 1.194 (0.870-1.639) | 1.096 (0.946-1.269) |
| Cerebral infarction, unspecific | 0.640 (0.181-2.271) | 0.651 (0.387-1.094) |
| Ischemic heart disease | 1.264 (0.753-2.121) | **0.664 (0.517-0.855)** |
| Three hypers | 1.287 (0.976-1.697) | 1.110 (0.987-1.248) |
| **All-cause mortality** | 1.279 (0.958-1.706) | 0.854 (0.629-1.161) |

Note:

ULTs: Urate-lowering therapeutics, CI: Confidence interval. NA: Not available.

a. Propensity score matching was performed on all listed characteristics.
b. Urate ≦ 5 mg/dL occurred within 6 months after the index date.
c. After the index date, there were no occurrences of urate levels below 5 mg/dl within the following six months.

Table S11. Sensitivity analysis_ duration between hyperuricemia and ULTs prescription

|  | **Adjusted hazard ratio (95% CI) ^a^** | | |
| --- | --- | --- | --- |
| **Outcomes**  **(**ULTs cohort vs. Control cohort) | **Within 30 days^b^**  (n= 2077 pairs) | **31 days to 90 days^c^**  (n= 265 pairs) | **More than 91 days^d^**  (n= 631 pairs) |
| **Composite outcome** | **1.292 (1.146-1.456)** | **1.423 (1.018-1.989)** | 1.055 (0.859-1.296) |
| Essential Hypertension | **1.219 (1.048-1.418)** | 1.447 (0.957-2.189) | 1.113 (0.854-1.451) |
| Cerebral infarction, unspecific | 0.621 (0.355-1.086) | 0.551 (0.131-2.310) | 1.012 (0.469-2.183) |
| Ischemic heart disease | 0.797 (0.621-1.022) | 0.723 (0.381-1.370) | 0.883 (0.570-1.370) |
| Three hypers | **1.274 (1.129-1.438)** | 1.337 (0.954-1.874) | 1.040 (0.846-1.279) |
| **All-cause mortality** | **1.859 (1.498-2.307)** | 0.729 (0.345-1.542) | 0.672 (0.401-1.127) |

Note:

ULTs: Urate-lowering therapeutics, CI: Confidence interval. NA: Not available.

a. Propensity score matching was performed on all listed characteristics.
b. ULTs cohort were those prescribed ULTs within 30 days after 1^st^ hyperuricemia.
c. ULTs were prescribed within 31~90 days after 1^st^ hyperuricemia and cannot have ULTs within 30 days after 1^st^ hyperuricemia.

d. ULTs were prescribed 91 days after 1^st^ hyperuricemia and cannot have ULTs within 90 days after 1^st^ hyperuricemia.
